# Recognition of Facial Expressions of Different Emotional Intensities in Patients with Frontotemporal Lobar Degeneration

**DOI:** 10.1155/2007/868431

**Published:** 2007-02-12

**Authors:** Roy P. C. Kessels, Lotte Gerritsen, Barbara Montagne, Nibal Ackl, Janine Diehl, Adrian Danek

**Affiliations:** ^1^Department of Experimental PsychologyHelmholtz InstituutUtrecht UniversityThe Netherlands; ^2^Department of NeurologyUniversity Medical CenterUtrechtThe Netherlands; ^3^Department of PsychiatryRadboud University Medical Centre NijmegenThe Netherlands; ^4^Neurologische Klinik und PoliklinikLudwig-Maximilians-Universität MünchenGermany; ^5^Klinik und Poliklinik für Psychiatrie der TU MünchenGermany

**Keywords:** Frontotemporal lobar degeneration, dementia, emotion, facial expression, face recognition

## Abstract

Behavioural problems are a key feature of frontotemporal lobar degeneration (FTLD). Also, FTLD patients show impairments in emotion processing. Specifically, the perception of negative emotional facial expressions is affected. Generally, however, negative emotional expressions are regarded as more difficult to recognize than positive ones, which thus may have been a confounding factor in previous studies. Also, ceiling effects are often present on emotion recognition tasks using full-blown emotional facial expressions. In the present study with FTLD patients, we examined the perception of sadness, anger, fear, happiness, surprise and disgust at different emotional intensities on morphed facial expressions to take task difficulty into account. Results showed that our FTLD patients were specifically impaired at the recognition of the emotion anger. Also, the patients performed worse than the controls on recognition of surprise, but performed at control levels on disgust, happiness, sadness and fear. These findings corroborate and extend previous results showing deficits in emotion perception in FTLD.

## 1. Introduction

Frontotemporal lobar degeneration (FTLD) is a neurodegenerative disorder characterized by atrophy of the frontal cortex and the anterior parts of the temporal lobes, and is a relatively common cause of dementia, accounting for about twenty percent of cases of dementia with pre-senile onset [14]. The earliest neurobehavioural changes in FTLD often relate to changes in personality and social conduct. Clinical findings include loss of insight, reduced empathy, poor self care, mood changes, changes in eating pattern, disinhibition, apathy and mental rigidity [18]. Furthermore, a deficit in emotion processing may represent a core problem in patients with FTLD [5], since the neuropathology involves circuits that are highly important in emotion processing, such as orbitofrontal cortex, amygdala and temporal cortex [1,2].

A number of studies have recently examined emotion processing in patients with FTLD. Lavenu et al. [7] were the first to examine a group of patients on recognition of facial expressions and used the seven emotions anger, sadness, disgust, happiness, fear, surprise and contempt. Their patients performed worse than healthy controls and patients suffering from Alzheimer's disease (AD) on the emotions anger, sadness and disgust. A recent follow-up study showed that in about

ISSN 0953-4180/07/$17.00 © 2007 – IOS Press and the authors. All rights reserved half of the patients with FTLD, emotion perception worsened with disease progression [8]. This finding was replicated by Fernandez-Duque et al. [5] who also showed that FTLD patients performed worse on negative emotions than controls and AD patients. In addition, a distinction can be made between FTLD patients with predominantly frontal atrophy (frontal variant) and patients with predominantly temporal lobe pathology (temporal variant). The two types of FTLD patients have been demonstrated to have equal difficulty in recognizing negative emotions, but the patients with the frontal variant also displayed impaired recognition of happiness [15]. These deficits in FTLD have been linked to pathology especially in the right inferior temporal lobe including the amygdala [16,17]. There is additional evidence that emotion recognition deficits are supramodal, since FTLD patients showed impaired perception of vocal emotions as well [6]. Clinically, these findings may be useful in the assessment of patients in whom FTLD is suspected. Moreover, the performance on emotion perception tasks may aid to follow the course of the disease [16].

Until now, most studies on facial expression recognition in FTLD have used static photographs of actors that mimic full-blown facial expressions, such as the Ekman and Friesen stimuli [4]. However, it has been suggested that the finding of a specific FTLD impairment in negative emotions was due to differences in task difficulty. That is, negative emotions are generally less easily recognized than positive emotions, even by healthy subjects [5]. Possibly, a ceiling effect may explain the intact recognition of positive emotions in previous FTLD studies. In the present study, morphed dynamic facial expressions (i.e., video clips) of different emotional intensities were used to investigate different levels of difficulty across emotion types. That is, a full-blown emotion, such as happiness at 100% intensity, may be easy to recognize even by patients with cognitive impairment, but lowering the intensity of the emotion increases the difficulty, making them better comparable with the other emotion types. By examining the performance of FTLD patients on this paradigm, more subtle emotion deficits may possibly be identified that have not been detected in previous studies using static images, which are probably less sensitive. If task difficulty indeed plays a role in the performance of FTLD patients on emotion perception, it can be hypothesised that FTLD patients are impaired at all emotion types at lower intensities, compared to age-matched controls. Alternatively, it may be expected that the present paradigm results in selective impairments in the perception of negative emotions, especially anger, sadness and disgust, that are not due to differences in difficulty across emotion types.

## 2. Methods

### *2.1. Participants*

Ten FTLD patients (4 males) with a mean age of 64.5 ($SD = 7.9$, range 43–74) were recruited from the neurology department of Ludwig-Maximilians-Universität Munich and the psychiatric clinic of Technische Universität Munich. The diagnosis of FTLD was based on the results of neuropsychological testing, laboratory investigations and on behavioural observation in accordance with the Neary criteria [14]. Neuroimaging data (MRI and PET where available) supported the clinical diagnosis; four patients had the frontal variant, three the temporal variant and three patients had a mixed frontotemporal atrophy pattern. Mean duration of illness was 3.8 years ($SD = 2.0$). Mean performance on the Mini-Mental State Examination (MMSE) was 25.0 ($SD = 2.16$; range 22–28). Thirty-eight healthy subjects (23 males) served as controls, with a mean age of 59.5 ($SD = 8.1$, range 47–75). None of the control participants had a history of neurological or psychiatric disease. Written informed consent was obtained according to the declaration of Helsinki. There were no group differences between patients and controls for distribution of age ($t(46) = 1.7$, $p = 0.26$) or gender (male vs. female, patients vs. controls: Mann-Whitney $U = 189.0$, $Z = -0.03$, $p = 0.98$).

### *2.2. Materials and procedure*

#### *2.2.1. Neuropsychological testing*

All patients were examined using the CERAD Neuropsychological Battery [13] that incorporates the MMSE (scoring range 0–30) and included the following subtests: Verbal Fluency (animal naming, 60 s, scoring range 0 on up), Modified Boston Naming Test (15 items; scoring range 0–15), Word List Learning (10 items in 3 trials, scoring range 0–30), Constructional Praxis (copying circle, diamond, two rectangles and cube, scoring range 0–11), Word List Recall (recall of previously learned words, scoring range 0–10), Word List Recognition (recognition of previously learned words from ten new distracter items, scoring range 0–20). An individual test performance was regarded as "impaired" if more than 2 standard deviations below the mean of the normative sample [19]. Table 1 shows the CERAD performance for the FTLD patients.

Table 1
Test results for the FTLD group on the CERAD battery [19]

| Neuropsychological test | Mean | SD | Number of impaired patients |
|---|---|---|---|
| MMSE | 25.0 | 2.2 | 5 |
| Verbal Fluency | 6.6 | 2.6 | 10 |
| Modified Boston Naming Test | 9.3 | 3.9 | 5 |
| Word List Learning | 13.1 | 4.7 | 4 |
| Word List Recall | 3.1 | 2.3 | 8 |
| Word List Recognition | 15.4 | 3.5 | 2 |
| Constructional Praxis | 6.7 | 3.5 | 6 |

Table 2
Number of correct responses for the FTLD patients and the controls (means and standard deviations, SD) on the facial expressions with 100% emotional intensity (maximum score per emotion = 4)

| | FTLD patients | | Controls | | |
|---|---|---|---|---|---|
| | Mean | SD | Mean | SD | |
| Anger | 1.7 | 1.2 | 2.9 | 1.1 | $p < 0.005$ |
| Disgust | 1.8 | 1.9 | 2.9 | 1.1 | n.s. |
| Fear | 0.6 | 0.8 | 1.3 | 1.1 | n.s. |
| Happiness | 3.6 | 0.8 | 3.9 | 0.5 | n.s. |
| Sadness | 1.0 | 0.9 | 1.7 | 1.0 | n.s. |
| Surprise | 1.5 | 1.1 | 2.4 | 1.3 | n.s. |

### *2.2.2. Emotion recognition task*

This task [11,12] was developed to measure the perception of facial emotional expressions using the six universal emotions anger, sadness, surprise, happiness, fear and disgust [10]. A computer-generated program was devised to enable a real-time interactive morphing between two endpoint facial expressions (0% emotion, i.e., neutral, and 100% emotion, i.e. anger, happiness, sad, fear, surprise, disgust) of the same identity [3]. Each morph was made with 21 images between 0% and 100% that were used to construct video clips that incrementally increased the degree of expression by 10% steps, starting at a 20% intensity level (see Fig. 1 for an example). The video clips were presented beginning with the lowest intensities (i.e., first all video clips showing expressions changing from neutral into 20% emotion, subsequently all clips changing from neutral into 30% emotions, and so on until the full-blown 100% expressions). Following three practice trials, the different types of emotion of the same intensity were presented in pseudo-random but fixed order to control for possible order effects of previously encountered emotion types that may influence the response [18]. In each trial, subjects had to indicate which expression was being presented by pointing at one of the six text-labelled icons (i.e., forced choice). The duration of the video clips was approximately 1 to 3 seconds, depending on the number of steps. The performance on each trial was recorded (i.e. correct/incorrect).

### *2.3. Analyses*

First, in order to compare the data of the present study with previous findings, the performance on the 100% emotion trials was analysed using t-tests for each emotion type with group as between-subject factor (Bonferroni-corrected for multiple comparisons, $\alpha = 0.0083$). Subsequently, data were analyzed using a multivariate general linear model (GLM) repeated-measures analysis for the Emotion Recognition Task, with Emotion Type (6 levels: anger, happiness, surprise, fear, sadness and disgust) and Emotion Intensity (9 levels: 20%–100%) as within subject factors and Group (patient vs. control) as between-subject variable. Subsequently, Bonferroni-corrected ($\alpha = 0.0083$) repeated-measures GLM analyses were performed for the six emotions separately (with Emotion Intensity as within-subject factor and Group as between-subject factor).

## 3. Results

Table 2 shows the performance of both groups on the 100% emotion trials (i.e., morphs changing from a neutral expression to 100% emotional intensity) for the six emotions. The FTLD patients performed worse than the controls on the emotion anger ($t(46) = 2.97$, $p < 0.005$). No significant group differences were found on any of the other emotions (all t-values <2.26). Figure 2 shows the performance on the nine levels of emotional intensities (20–100%) for the six emotions. Overall, main effects were found for Emotion Type ($F(5, 42) = 62.7$, $p < 0.0005$), Emotion Intensity ($F(8, 39) = 10.1$, $p < 0.0005$) and Group ($F(1, 46) = 15.2$, $p < 0.0005$). This indicates that the performance improved when faces with a higher level of emotion intensity were presented. Overall, the FTLD patients performed worse than the controls. Additionally, an Emotion Type $\times$ Group interaction effect was found ($F(5, 42) = 2.8$, $p < 0.03$), showing that the impairments of the FTLD group was not equal for all emotions. Subsequent analyses for the six emotion types were performed separately, showing a Group effect for Anger ($F(1, 46) = 19.4$; $p < 0.0005$) and Surprise ($F(1, 46) = 10.3$, $p < 0.002$), but not for Sadness ($F(1, 46) = 1.5$), Disgust ($F(1, 46) = 3.7$), Fear ($F(1, 46) = 1.7$) and Happiness ($F(1, 46) = 3.9$). Significant main effects for intensity were found for all emotions (all F-values $> 3.1$, $p < 0.009$), except for the emotion Fear ($F(8, 39) = 1.8$). None of the Group $\times$ Emotion Intensity interactions was significant (all F-values $< 2.4$).

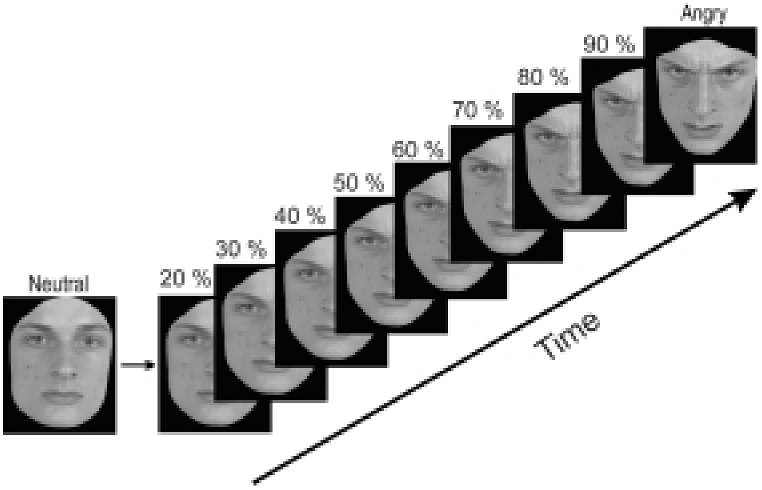

Fig. 1. The Emotional Recognition Task. Picture shows the nine end points of emotional intensity for the angry face. In the actual test, video clips were shown morphing from neutral to one of these end points for all emotion types.

## 4. Discussion

The aim of the present study was to examine the perception of emotional facial expressions in patients with FTLD. The results on the full-blown facial expressions showed that the FTLD patients were impaired at the recognition of the emotion anger, but performed equal to controls on the other emotions. This is in agreement with a recent study in which morphed emotional facial expressions (from neutral to the full-blown emotion) were presented to FTLD patients and controls [9]. These patients also performed more poorly than controls on the full-blown emotion anger, whereas, when corrected for multiple comparisons, the performance on the other full-blown emotion types did not significantly differ from the control performance. Furthermore, in that study the data on sensitivity (perception of the expressions at lower intensities) could not be analysed. Although participants were prompted to name the target emotion as quickly as possible during the presentation of the morphs with increasing emotional intensity, the patients performed at floor level: they did not name the emotion until the presentation was completed [9]. Since patients with frontotemporal dementia show impairments in speed of information processing compared to healthy controls [21], timed tasks disproportionally hamper the performance in these patients. In the present study, we therefore did not investigate reaction times, but presented different video clips that morphed from neutral into different intensity levels for the separate emotions, after which patients responded. Additionally, participants had to respond by selecting one out of six emotions, that is, a forced choice paradigm was used in our study rather than naming.

The trials with different end points of emotional intensity demonstrated that the FTLD patients performed worse than the controls on anger and surprise, but did not differ from healthy age-matched controls on disgust, happiness, sadness, surprise and fear. In addition, while the patients had more difficulty overall in correctly recognizing emotions at lower intensities compared to full-blown facial expressions, this difficulty was equally present in the controls. Thus, the present results corroborate and extend previous findings showing a selective deficit in emotion perception, indicating that previous results were reliable and not due to differences in task difficulty. The finding that FTLD patients were specifically impaired at the recognition of anger is again in agreement with previous findings [5,7,9,15]. Impaired surprise recognition has been demonstrated in some patients with FTLD [6]. Furthermore, it was demonstrated that recognition of surprise substantially decreased with disease progression and that FTLD patients performed even worse than AD patients on this emotion [8]. However, more studies are needed to replicate this result.

Previous studies have also reported deficits on sadness [5,7,15] and disgust [5,7,9], while our patients performed equally to controls. However, a follow-up study of Lavenu et al. [8] showed that the recognition of sad emotional expressions, although impaired at initial testing [7], actually improved with progression of the disease, both in FTLD patients as well as in AD. It

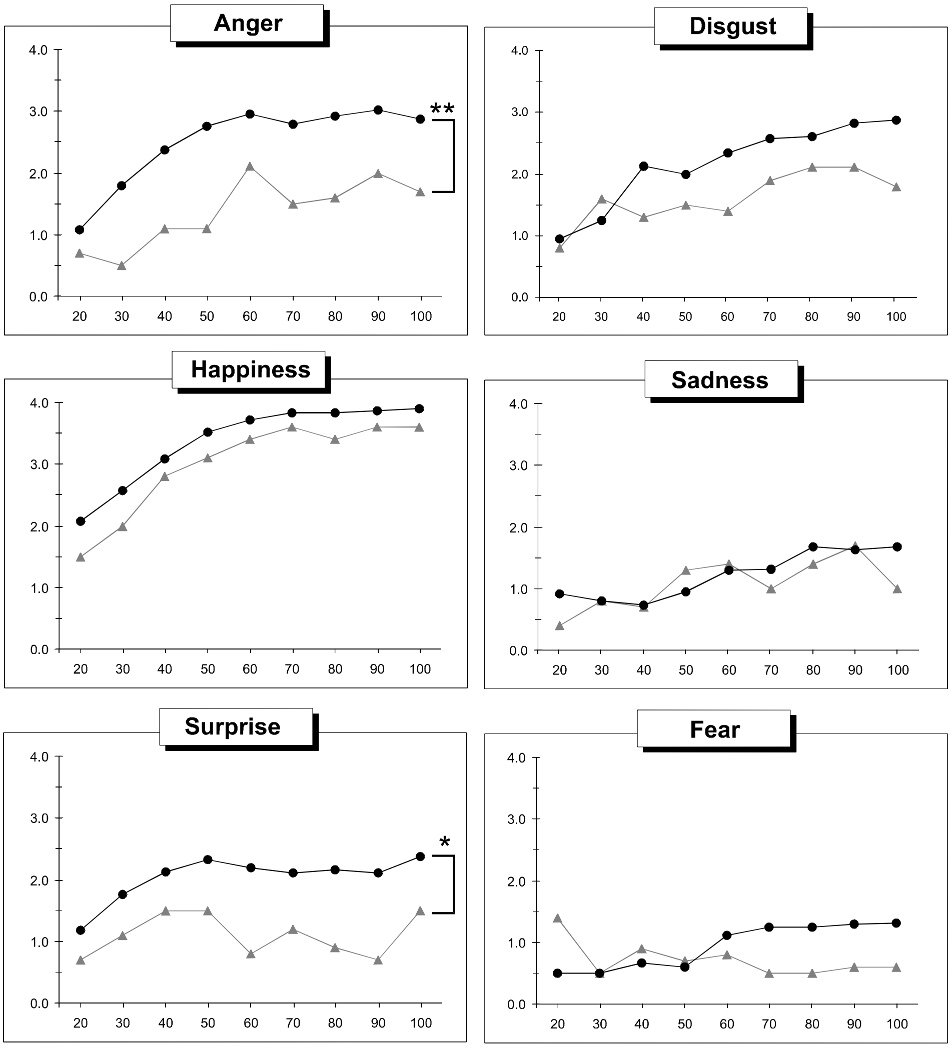

Fig. 2. Mean performance (y-axis, number correctly identified emotions) of the FTLD patients (grey triangles) and the healthy controls (black circles) on nine end points of emotional intensity (x-axis, 20–100%) for the six different emotions (maximum score for each intensity per emotion type = 4); Levels of significance: $*p < 0.002$, $**p < 0.0005$.

should be noted, however, that their sample consisted of only six FTLD patients. In turn, Rosen et al. [17] showed that impairments in the recognition of sadness were correlated with atrophy in the temporal cortex, but this conclusion was not only based on FTLD patients, but also on patients with AD who generally show profound temporal-lobe atrophy [20]. In line with previous results, we did not find an impaired performance on the emotion fear [6,7]. In some studies though, FTLD patients have been found to be impaired at the recognition of fearful emotional expressions as well [15,16]. These mixed results may be due to task difficulty, in that fearful facial expressions are regarded as difficult by healthy participants as well and thus may be most susceptible to cognitive decline due to a neurodegenerative disease.

Differences in patient characteristics or brain pathology may also explain the variability of emotion recognition in FTLD. For example, impairment in disgust recognition in FTLD has specifically been linked to atrophy of the insularcortex [9]. Due to the small sample size, we unfortunately could not systematically compare patients with frontal variant and temporal variant FTLD. Future studies should focus in more detail on the neural substrates involved in emotion recognition. Additionally, with respect to cognitive functioning in general, patients performed only a limited neuropsychological test battery, the CERAD battery, which mainly focuses on verbal functions. Thus, it was not possible to relate the deficits in emotion perception to the performance in other cognitive domains in which FTLD patients often show impairments, such as executive functioning. Future studies should address in more detail the relation between emotion perception and other cognitive functions.

In summary, the present study is the first to examine the perception of dynamic morphed facial expressions in FTLD patients without restrictions in response time. The results demonstrated that FTLD patients display a robust impairment in recognizing the emotion anger and agree with previous findings. We also demonstrated a deficit in recognizing the emotion surprise. Clearly, the current paradigm is sensitive to demonstrate selective impairments in FTLD, even in small sample sizes. In the clinical assessment of individual patients, it may be useful to include measures of emotion perception, along with other tasks sensitive to frontotemporal pathology.

## Acknowledgements

The authors would like to thank Gisela Stenglein-Krapf, Vilma Serrone and Thomas Göhringer for their assistance with the neuropsychological tests. Barbara Montagne was supported by grant #490-22-203 from the Netherlands Organization for Scientific Research (NWO).

## References

[1] R. Adolphs, Neural systems for recognizing emotion, *Current Opinion in Neurobiology* **12** (2002), 169–177.

[2] R. Adolphs, H. Damasio, D. Tranel and A.R. Damasio, Cortical systems for the recognition of emotion in facial expressions, *Journal of Neuroscience* **1** (1996), 7678–7687.

[3] P.J. Benson and D.I. Perrett, Synthesising continuous-tone caricatures, *Image and Vision Computing* **9** (1991), 123–129.

[4] P. Ekman and W.V. Friesen, *Unmasking the face*, Englewood Cliffs, NJ, Prentice Hall, 1975.

[5] D. Fernandez-Duque and S.E. Black, Impaired recognition of negative facial emotions in patients with frontotemporal dementia, *Neuropsychologie* **43** (2005), 1673–1687.

[6] J. Keane, A.J. Calder, J.R. Hodges and A.W. Young, Face and emotion processing in frontal variant frontotemporal dementia, *Neuropsychologia* **40** (2002), 655–665.

[7] I. Lavenu, F. Pasquier, F. Lebert, H. Petit and M. Van der Linden, Perception of emotion in frontotemporal dementia and Alzheimer's disease, *Alzheimer Disease and Associated Disorders* **13** (1999), 96–101.

[8] I. Lavenu and F. Pasquier, Perception of emotion on faces in frontotemporal dementia and Alzheimer's disease: a longitudinal study, *Dementia and Geriatric Cognitive Disorders* **19** (2005), 37–41.

[9] S. Lough, C.M. Kipps, C. Treise, P. Watson, J.R. Blair and J.R. Hodges, Social reasoning, emotion and empathy in frontotemporal dementia, *Neuropsychologia* (2005), in press.

[10] M.K. Mandal and N. Ambady, Laterality of facial expressions of emotion: universal and culture-specific influences, *Behavioural Neurology* **15** (2004), 23–34.

[11] B. Montagne, R.P.C. Kessels, M.P.M. Kammers, E. Kingma, E.H.F. de Haan, R.A.C. Roos and H.A.M. Middelkoop, Perception of emotional facial expressions at different intensities in early-symptomatic Huntington's Disease, *European Neurology* **55** (2006), 151–154.

[12] B. Montagne, R.P.C. Kessels, E. Frigerio, E.H.F. de Haan and D.I. Perrett, Sex differences in the perception of affective facial expressions: do men really lack emotional sensitivity? *Cognitive Processing* **6** (2005), 136–141.

[13] J.C. Morris, A. Heyman, R.C. Mohs, J.P. Hughes, G. van Belle, G. Fillenbaum, E.D. Mellits and C. Clark, The Consortium to Establish a Registry for Alzheimer's Disease (CERAD). Part I. Clinical and neuropsychological assessment of Alzheimer's disease, *Neurology* **39** (1989), 1159–1165.

[14] D. Neary, J.S. Snowden, L. Gustafson, U. Passant, D. Stuss, S. Black, M. Freedman, A. Kertesz, P.H. Robert, M. Albert, K. Boone, B.L. Miller, J. Cummings and D.F. Benson, Frontotemporal lobar degeneration: a consensus on clinical diagnostic criteria, *Neurology* **51** (1998), 1546–1554.

[15] H.J. Rosen, K. Pace-Savitsky, R.J. Perry, J.H. Kramer, B.L. Miller and R.W. Levenson, Recognition of emotions in the frontal and temporal variants of frontotemporal dementia, *Dementia and Geriatric Cognitive Disorders* **17** (2004) 277–281.

[16] H.J. Rosen, R.J. Perry, J. Murphy, J.H. Kramer, P. Mychack, N. Chuff, M. Weiner, R.W. Levenson and B.L. Miller, Emotion comprehension in the temporal variant of frontotemporal dementia, *Brain* **125** (2002), 2286–2295.

[17] H.J. Rosen, M.R. Wilson, G.F. Schauer, S. Allison, M.L. Gorno-Tempini, C. Pace-Savitsky, J.H. Kramer, R.W. Levenson, M. Weiner and B.L. Miller, Neuroanatomical correlates of impaired recognition of emotion in dementia, *Neuropsychologia* **44** (2006), 365–373.

[18] J.S. Snowden, D. Neary and D.M.A. Mann, *Fronto-temporal lobar degeneration: fronto-temporal dementia, progressive aphasia, semantic dementia*, Churchill Livingstone, New York, 1996.

[19] B. Thalmann , A.U. Monsch, M. Schneitter, D. Ermini-Fünfschilling, R. Spiegel and H.B. Stähelin, *Die CERAD Neuropsychologische Testbatterie: Ein gemeinsames minimales Instrumentarium zur Demenzabklärung*, Memory Clinic, Basel, http://www.memoryclinic.ch/tests/index.php, 1998.

[20] W.M. van der Flier, H.A.M. Middelkoop, A.W. Weverling-Rijnsburger, F. Admiraal-Behloul, A. Spilt, E.L.E.M. Bollen, G.G.J. Westendorp and M.A. van Buchem, Interaction of medial temporal lobe atrophy and white matter hyperintensities in AD, *Neurology* **62** (2004), 1862–1864.

[21] A.J. Walker, S. Meares, P.S. Sachdev and H. Brodaty, The differentiation of mild frontotemporal dementia from Alzheimer's disease and healthy aging by neuropsychological tests, *International Psychogeriatrics* **17** (2005), 57–68.
